# Crop Classification Based on Red Edge Features Analysis of GF-6 WFV Data

**DOI:** 10.3390/s21134328

**Published:** 2021-06-24

**Authors:** Yupeng Kang, Qingyan Meng, Miao Liu, Youfeng Zou, Xuemiao Wang

**Affiliations:** 1 School of Surveying and Land Information Engineering, Henan Polytechnic University, Jiaozuo 454003, China; kangyphpu_x86@home.hpu.edu.cn (Y.K.); zouyf@hpu.edu.cn (Y.Z.); 2 Aerospace Information Research Institute, Chinese Academy of Sciences, Beijing 100101, China; liumiao@aircas.ac.cn (M.L.); wangxuemiao19@mails.ucas.ac.cn (X.W.); 3 Sanya Institute of Remote Sensing, Sanya 572029, China

**Keywords:** GF-6 WFV data, red edge features, crop classification, spectral analysis, red edge indices, feature evaluation

## Abstract

A red edge band is a sensitive spectral band of crops, which helps to improve the accuracy of crop classification. In view of the characteristics of GF-6 WFV data with multiple red edge bands, this paper took Hengshui City, Hebei Province, China, as the study area to carry out red edge feature analysis and crop classification, and analyzed the influence of different red edge features on crop classification. On the basis of GF-6 WFV red edge band spectral analysis, different red edge feature extraction and red edge indices feature importance evaluation, 12 classification schemes were designed based on GF-6 WFV of four bands (only including red, green, blue and near-infrared bands), stepwise discriminant analysis (SDA) and random forest (RF) method were used for feature selection and importance evaluation, and RF classification algorithm was used for crop classification. The results show the following: (1) The red edge 750 band of GF-6 WFV data contains more information content than the red edge 710 band. Compared with the red edge 750 band, the red edge 710 band is more conducive to improving the separability between different crops, which can improve the classification accuracy; (2) According to the classification results of different red edge indices, compared with the SDA method, the RF method is more accurate in the feature importance evaluation; (3) Red edge spectral features, red edge texture features and red edge indices can improve the accuracy of crop classification in different degrees, and the red edge features based on red edge 710 band can improve the accuracy of crop classification more effectively. This study improves the accuracy of remote sensing classification of crops, and can provide reference for the application of GF-6 WFV data and its red edge bands in agricultural remote sensing.

## 1. Introduction

Crop classification is an important part of agricultural remote sensing monitoring, as well as the basis and key link of the application of remote sensing technology in the field of agriculture [1]. Timely and accurate classification of crops by remote sensing can determine planting area, spatial distribution, landscape pattern and planting structure of crops accurately, and provide important input parameters for crop yield estimation and growth monitoring [1,2,3]. The study on crop classification by remote sensing is of great significance for guiding agricultural production, formulating agricultural policy, ensuring food security and realizing sustainable agricultural development [4,5].

With the continuous development of remote sensing science and technology, moderate-to-high spatial resolution remote sensing data have been widely used in land use and land cover classification, crop classification, and so on [6,7,8,9]. The traditional moderate-to-high spatial resolution multispectral optical satellite payload is mainly composed of four bands: blue (450–520 nm), green (520–590 nm), red (630–690 nm) and near-infrared (770–890 nm). The limited bands make it difficult to meet the requirements of fine classification of crops by remote sensing. The red edge band is between the red band and the near-infrared band, and the band range is roughly 670–780 nm. The red edge is the region where the spectral reflectance of green vegetation rises rapidly within a certain band range, and it is the sensitive spectral band of vegetation, which is closely related to the pigment state and physical and chemical properties of crops and other vegetation [10]. Red edge information was first used in hyperspectral remote sensing, which is often used in the inversion of vegetation physiological and biochemical parameters such as chlorophyll content, leaf area index (LAI), biomass, nitrogen content, crop growth and pest monitoring, etc. [11,12,13,14]. In addition, some studies have shown that the red edge band can enhance the separability between different ground features, which plays an important role in improving the accuracy of crop remote sensing classification [15,16].

Based on the recognition of the importance of red edge band, more and more multispectral satellite payloads have improved the application ability of remote sensing satellites by adding red edge bands, such as RapidEye satellite of Germany, WorldView-2/3 satellite of the United States, Sentinel-2 satellite of ESA and Gaofen-6 (GF-6) satellite of China. Many studies have confirmed that the addition of red edge bands can significantly improve the classification accuracy of crops [17,18,19,20]. Meanwhile, the red edge index, red edge texture and other features based on red edge bands enrich the feature space of crop classification, which is conducive to better use of different red edge features. For example, Kim et al. [21] used two seasons’ RapidEye images to classify rice crops in Jeonju, Korea, and applied the spectral and texture features of broadband red edge in different rice classification, which confirmed that broadband red edge information has the potential to improve the accuracy of rice classification. Ustuner et al. [22], based on RapidEye images, discussed the impact of three different vegetation indices on the classification accuracy of crops in the Aegean Sea area of Turkey, including normalized vegetation index (NDVI), green normalized difference vegetation index (GNDVI) and normalized difference edge index (NDRE), and found that NDRE contributed the most to classification accuracy. Huang et al. [23] adopted Sentinel-2A data of time series to extract crop classification information, by introducing parcel elements and improved chlorophyll absorption red edge index (MCARI), combined with machine learning methods, to explore the impact of different classification feature combinations on classification accuracy, which showed that the introduction of red edge spectra and red edge indices could significantly improve the identification ability of crops in arid areas. Wu et al. [24] selected the multitemporal Sentinel-2A remote sensing data, calculated the NDVI and red edge normalized vegetation index (RENDVI), designed five different vegetation indices time series combination feature classification schemes, and used the random forest algorithm to realize the fine classification of crops. The classification results prove that the red edge indices can assist NDVI to improve the classification accuracy.

At present, machine learning is widely used in the research of crop remote sensing classification algorithm. There are many kinds of machine learning classification methods, such as support vector machine (SVM), random forest (RF), decision tree (DT), maximum likelihood (ML), k-nearest neighbor (k-NN) and artificial neural network (ANN), which can be applied to the research of land cover and land use classification, crop classification and other remote sensing applications, among which random forest is one of the most commonly used machine learning classification methods [25]. Zeraatpasheh et al. [26] used a variety of linear and nonlinear machine learning algorithms, including Cubist (Cu), random forest (RF), regression tree (RT) and multiple linear regression (MLR), to conduct a digital mapping of soil properties in the semi-arid Borujen region of central Iran. Sonobe et al. [27] compared kernel-based extreme learning machine (KELM), multilayer feedforward neural network (FNN), random forest (RF) and support vector machine (SVM) using Sentinel-1A and Sentinel-2A data, and evaluated the sensitivity of the different supervised learning models in the study area of Hokkaido, Japan. Maponya et al. [28] evaluated the classification performance of SVM (support vector machine), DT (decision tree), k-NN (k-nearest neighbor), RF (random forest) and ML (maximum likelihood) for different time series Sentinel-2 data in two different sites in the Western Cape, South Africa, and concluded that SVM and RF can obtain better classification accuracy and greater application potential. In a word, compared with different machine learning classification methods, RF classification method has the characteristics of less parameter setting, stability, maturity and high classification accuracy, so it is suitable for the classification and comparative study of different red edge features in this study.

Although the current research generally confirms the important role of the red edge band for crop classification, most studies only use different red edge spectra or red edge indices participating in the classification to test its effect on crop classification, which is not enough for the analysis and evaluation of different red edge features, especially for GF-6 WFV, Sentinel-2 and other remote sensing satellites with multiple red edge bands. To resolve these problems, this study is mainly aimed at: (1) Performing spectral feature analysis and red edge indices feature importance evaluation of GF-6 WFV data, and extracting different red edge spectra, red edge textures and red edge indices; (2) Designing red edge feature classification schemes for crop classification in Hengshui City, analyzing the impact of different red edge features on crop classification, improving the accuracy of crop classification and promoting the application of GF-6 WFV data in the field of agricultural remote sensing.

## 2. Study Area and Data Sources

### 2.1. Overview of the Study Area

The study area is Hengshui City, located in the southeast of Hebei Province, China, with a total area of 8815 km^2^. Hengshui City belongs to the semi-humid and semi-arid monsoon climate zone in the warm temperate zone, with four distinct seasons, sufficient sunshine and annual average precipitation of 500–600 mm. The study area has rich land resources and diverse soil types, which are suitable for planting and growing a variety of crops, and the coverage rate of crops is more than 70%. The crop types in Hengshui City include winter wheat, summer maize, spring maize, cotton, fruit trees, greenhouse vegetables, etc. The main planting mode is winter wheat–summer maize rotation, two crops a year, and spring maize, cotton and other crops once a year [29,30]. The Heilonggang River Basin in Hebei Province, where the study area is located, is a typical groundwater funnel area in China and one of the pilot areas of the national fallow system. In recent years, affected by the national fallow policy and the transfer of rural labor, the planting area of winter wheat with large water consumption has decreased, while the planting proportion of spring maize has increased year by year [31]. Therefore, the study of crop classification by remote sensing is helpful to timely and accurately monitor the crop planting structure in Hengshui City, and is of great significance to implementing the national fallow policy and promoting the sustainable development of agriculture [32].

### 2.2. Data Source

#### 2.2.1. Remote Sensing Data

The GF-6 satellite is equipped with a 2 m panchromatic/8 m multi-spectral (PMS) camera and a 16 m multispectral wide-field-viewing (WFV) camera, which has the characteristics of high resolution and wide coverage, and the width of its wide-field-viewing camera can reach 800 km [33]. The GF-6 satellite was successfully launched at the Jiuquan Satellite Launch Center on 2 June 2018. For the first time in China, the wide-field camera on the GF-6 satellite has added two “red edge” bands that can effectively reflect the specific spectral characteristics of crops. At the same time, it will operate in a network with the GF-1 satellite in orbit, which can greatly improve China’s ability to monitor agriculture, forestry and other resources [34]. The main information parameters of GF-6 WFV data are shown in Table 1. In this paper, the GF-6 WFV image covering Hengshui City in the study area was selected on 28 August 2019. At that time, the crops in the study area were in a period of vigorous growth, which was suitable for remote sensing identification and classification research of crops. In order to meet the application requirements of crop classification by remote sensing, GF-6 WFV data were preprocessed by orthorectification, atmospheric correction and geometric correction to obtain surface reflectance data.

#### 2.2.2. Sample Data

**Figure 1:**
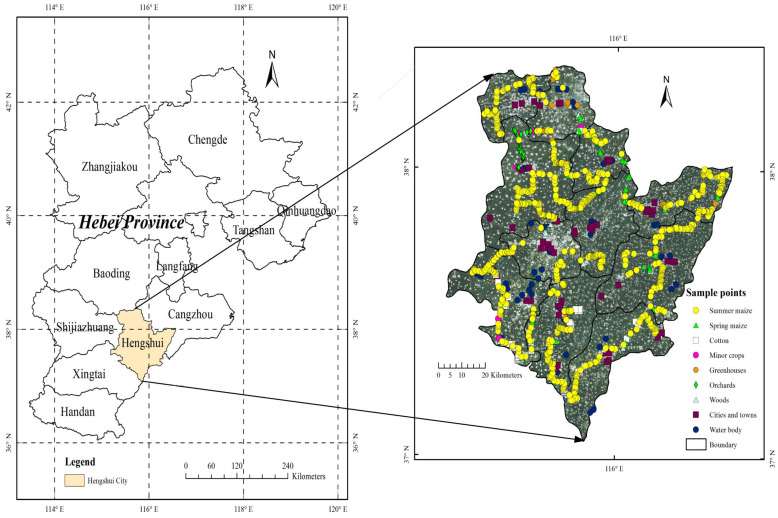
Geographical location and sample distribution of the study area.

The field sampling of different crops in Hengshui City was conducted in July 2019. At that time, the winter wheat had been harvested, and the summer maize with winter wheat and summer maize rotation system had basically sprouted in the stubble farmland, while the spring maize was in the jointing and heading stage, and the cotton flower was in the budding stage, which was conducive to the visual judgment of crop types. During the field survey, the sample points of different crop types in 11 counties and urban areas of Hengshui City were collected by handheld GPS, and photos were taken. The survey route covered most of the farmland in Hengshui City and the distribution was relatively uniform. A total of 614 valid sample plots were obtained in this survey, which were divided into training samples and validation samples according to the ratio of 1:1 for crop classification and accuracy evaluation. The geographical location and sample distribution of the study area are shown in Figure 1, and the number of samples for different classifications is shown in Table 2.

## 3. Methods

**Figure 2:**
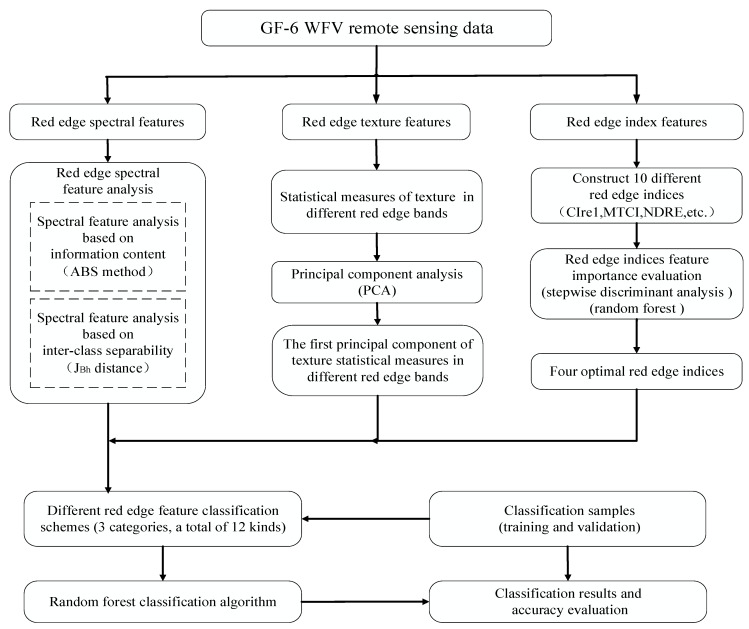
Technical route of the study.

The spectral features of remote sensing images and their derived features such as texture and vegetation index are the main basis of remote sensing image classification. GF-6 WFV data have several new spectral segments, and the feature analysis and optimization of these spectral segments is an important means of tapping into the potential of data application and ensuring classification accuracy. In this study, adaptive band selection (ABS) method and the extension of Jeffries–Matusita distance (J_Bh_) were used to analyze the spectral features of red edge bands of GF-6 WFV data based on information content and inter-class separability. Then, different red edge bands were used to construct the corresponding texture features and 10 different red edge index features. Stepwise discriminant analysis (SDA) and random forest (RF) feature importance evaluation algorithms were used to optimize the red edge indices. Finally, based on the spectrum, texture and optimal red edge index features of different red edge bands of GF-6 WFV data, different red edge feature classification schemes were designed, and the RF algorithm was used to classify crops according to different schemes. The technical route of the study is shown in Figure 2.

### 3.1. Spectral Feature Analysis

Since remote sensing classification mainly uses the spectra, texture and vegetation index features of the image, combined with the red edges and other new bands of GF-6 WFV data, it is very important to analyze the spectral features of GF-6 WFV data. In this study, the spectral feature analysis of GF-6 WFV data mainly adopts the ABS method based on information content and J_Bh_ distance method based on inter-class separability, so as to analyze the spectral feature of GF-6 WFV data from the two aspects of information content and inter-class separability.

#### 3.1.1. ABS Method

ABS is a sort-based band selection method based on the optimum index factor (OIF) mathematical model, which was proposed by Liu Chunhong et al. [35] on the basis of fully studying the OIF model. This method can select bands with rich information contents and little correlation with other bands. The calculation method of ABS method is shown in Equation (1), which is based on the following principles: (1) the selected band has more information content; (2) the selected band has less correlation with other bands [36]. The index obtained by the ABS method fully considers the similarity between the degree of information enrichment of each image and the adjacent bands. It has the characteristics of simple algorithm, convenient operation and effectively shortening the running time of the OIF model. In addition, the ABS method can obtain the evaluation index of a single band, which is beneficial to the spectral analysis and the study of remote sensing images in specific bands [37].

(1) $$I_{i}=\frac{\sigma_{i}}{\left(r_{i-1,i}+r_{i,i+1}\right)/2}$$

In the above equation, σ_i_ is the standard deviation of band I; $r_{i-1,i}$ and $r_{i,i+1}$ are the correlation coefficients between the band i and the two bands before and after it; $I_{i}$ represents the ABS index—the larger its value, the greater the information content in the corresponding band.

#### 3.1.2. J_Bh_ Distance

The performance of classifier classification largely depends on whether the feature can accurately describe the nature of the object. Therefore, a separability criterion such as Jeffries–Matusita (JM) distance is needed to measure the separability between different types of features. JM distance can better reflect the actual relationship with classification accuracy [38], but it can only measure the separability between each two classes and has the drawback that it cannot reflect the separability between multiple categories. Therefore, the J_Bh_ distance was introduced to measure the separability between multiple types of features [39]. Based on the Bhattacharyya principle, J_Bh_ distance gives greater weights to categories with higher probability of a priori, which is calculated according to the number of samples between different categories. The calculation method is shown in Equation (2):

(2) $$J_{Bh}=\sum_{i=1}^{M}\sum_{j>i}^{N}\sqrt{P\left(w_{i}\right)\times P\left(w_{j}\right)}\times JM^{2}\left(i,j\right)$$

In the above equation, N is the number of categories; $P(w_{i})$ and $P(w_{j})$ are the a priori possibilities of Class i and Class j, which are calculated according to the number of samples (pixels) [40].

### 3.2. Texture Feature Extraction

Texture feature is the law and feature formed by the repeated appearance of a large number of small objects in the image. It is a comprehensive reflection of the size, shape, shadow and color of a large number of individuals, and describes the spatial variation characteristics of pixel brightness. Gray-level co-occurrence matrix (GLCM) is a widely used method for extracting texture features. The commonly used statistical measures of texture features include Mean, Variance, Homogeneity, Contrast, Dissimilarity, Angular Second Moment (ASM), Entropy and Correlation, a total of 8 categories [41].

In this paper, 8 texture statistical measures with a 3 × 3 pixel-sized window were extracted from the two red edge bands and near-infrared band of GF-6 WFV data by GLCM method, respectively. Due to the redundancy between different texture statistical measures, the study will adopt the principal component analysis (PCA) method [42] to extract the first principal component (PC1) of the 8 texture statistical measures to represent the texture features of three different bands, so as to analyze the influence of the texture features of different red edge bands on crop classification.

### 3.3. Red Edge Index Analysis

Vegetation index is a linear or non-linear combination of spectral reflectance of remote sensing image, which can reflect some certain characteristics of vegetation information [43]. The red edge index is also known as the red edge vegetation index, namely, the vegetation index calculated by the surface reflectance of the red edge band. Since the red edge band is the sensitive characteristic spectral band of vegetation, the red edge index has an important influence on the classification of crops and other vegetation [44]. According to the characteristics of GF-6 WFV data with two red edge bands, this paper refers to relevant literature to construct 10 kinds of red edge indices for crop classification study [45,46]. The 10 red edge indices based on the GF-6 WFV data are shown in Table 3.

Feature selection and feature importance evaluation are of great significance for remote sensing image classification, which plays an important role in promoting model performance and improving classification model and algorithm [54]. Feature selection methods are divided into three categories: Filter, Wrapper and Embedded [55]. In this paper, the filter method of SDA and the embedded method of RF were used to evaluate 10 red edge vegetation indices of GF-6 WFV data [56,57]. In order to provide reference for the application of different red edge index features in crop classification, the importance of different red edge index features was analyzed, and the comparison and cross validation of SDA and RF were carried out.

### 3.4. Classification Scheme and Accuracy Evaluation

Based on the GF-6 WFV data of only red, green, blue and near-infrared bands, this paper designed some crop classification schemes by adding the spectra, texture and vegetation index features of one or two red edge bands to ensure that the feature dimensions involved in the classification are effectively consistent, so as to analyze the influence of the red edge 710, red edge 750 and near-infrared bands of GF-6 WFV data on crop classification [26,58]. The classification scheme design was first divided into three groups: A, B and C, according to the spectra, texture and vegetation index of different red edge bands, and then was subdivided into 12 specific classification schemes according to different combinations of red edge bands. The designs of different red edge feature classification schemes are shown in Table 4.

According to the texture features of different red edge bands, 8 texture statistical measures of GLCM were calculated for two red edge bands and a near-infrared band, respectively, and PCA was performed. The PC1 was extracted to form three texture features, and then four different texture feature classification schemes of group B were designed.

Based on two feature evaluation methods, namely SDA and RF, the first 4 red edge indices with high feature importance for classification were selected from the 10 red edge indices of GF-6 WFV data. Through different red edge indices participating in crop classification, the importance of different red edge indices and their impact on crop classification were analyzed, and the accuracy of different feature evaluation methods was verified, so as to provide reference for the application of different red edge indices in crop classification.

According to the abovementioned 12 red edge feature classification schemes, the RF classification module of EnMAP-Box Toolkit [59] was used to classify the main crop types in the study area. RF is a supervised classification method composed of multiple CART decision trees. It uses random resampling technology and node random splitting technology to construct multiple decision trees, and the final classification result is obtained through voting. Compared with traditional classification algorithms such as Maximum Likelihood Classification (MLC) and Support Vector Machine (SVM), the RF algorithm has a faster training speed and a higher degree of intelligence, is not easy to overfit and has high classification accuracy, and is widely used in crop classification and area statistics [60,61].

The evaluation of classification accuracy was based on the Confusion Matrix. The overall accuracy (OA), kappa coefficient, producer accuracy (PA) and user accuracy (UA) were selected to evaluate the accuracy of different classification schemes [62]. The F1 accuracy was used to evaluate the identification accuracy of a specific category of crops. F1 accuracy is the weighted harmonic mean of UA and PA [63], the specific calculation formula is shown in Equation (3):

(3) F1=2×UA×PA/(UA+PA)×100%

McNemar’s test was employed in this study to evaluate the statistical significance of the accuracy of the different classifiers or groups [64]. The test was based on the error matrix of two classifications and the chi-squared statistic value (χ^2^) with one degree of freedom was calculated as follows:

(4) $$\chi^{2}=\frac{{\left(f_{12}-f_{21}\right)}^{2}}{f_{12}{+f}_{21}}$$

where f_ij_ is the number of samples that are correctly classified by classification scheme i and incorrectly classified by classification scheme j (i = 1, 2; j = 1, 2). The difference between two classification schemes is statistically significant at the 95% confidence level (*p* = 0.05) when the χ^2^ value is greater than or equal to 3.84 [65,66]. In this study, McNemar’s test was used to evaluate whether there were significant differences in the classification accuracy between two classification schemes by RF classifier with different red edge features.

## 4. Results

### 4.1. Red Edge Spectral Analysis Results

#### 4.1.1. Spectral Analysis Based on Information Content

According to the characteristics of the ABS method, the GF-6 WFV data were first reconstructed with the purple band as the first band and the yellow band as the last band, so as to calculate and sort the ABS index of other bands of GF-6 WFV data. The ABS indices of GF-6 WFV data except the purple band and yellow band are shown in Table 5.

Combined with the results of the ABS index analysis, it can be concluded that the near-infrared band (NIR) and the two red edge bands (RE1, RE2) have more information content than the visible light bands (R, G, B), and the order of information content is as follows: NIR > RE2 > RE1 > R > G > B. The results show that the near-infrared and red edge bands of GF-6 WFV data can provide more information, which is beneficial to the classification of crops and other ground features.

#### 4.1.2. Spectral Analysis Based on Separability Distance

**Figure 3:**
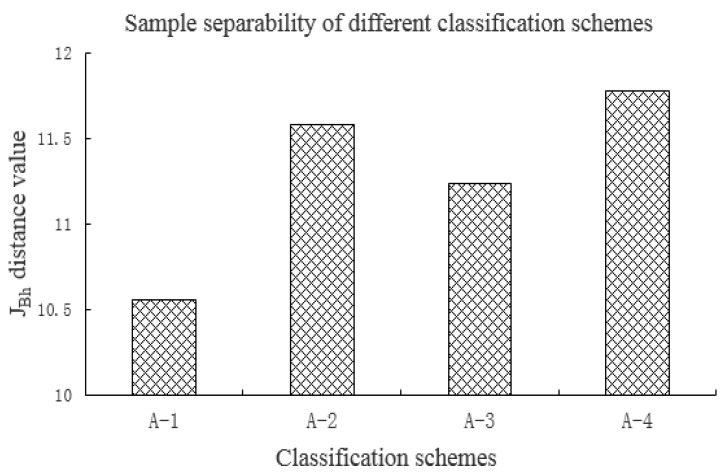
J_Bh_ distance of different spectral classification schemes for GF-6 WFV data.

According to the characteristics of GF-6 WFV data with two red edge bands, four different spectral classification schemes of “four bands”, “four bands + red edge 710”, “four bands + red edge 750” and “four bands + red edge 710 + red edge 750” were designed, corresponding to the classification schemes mentioned in Section 3.4, respectively. Combined with the training samples of different crops, the influence of different red edge bands on crop separability was analyzed by calculating J_Bh_ distance. J_Bh_ distance reflects the separability of crops in different classification schemes, and can be used to analyze the separability measure of different red edge bands of GF-6 WFV data to participate in crop classification. The J_Bh_ distance of four different classification schemes is shown in Figure 3.

The distances of schemes A-1, A-2, A-3 and A-4 were 10.561, 11.583, 11.239 and 11.777, respectively. The results show that the J_Bh_ distance of the two red edge bands (RE1, RE2) participating in the classification is the largest, the J_Bh_ distance of the red edge 710 band (RE1) is greater than the red edge 750 band (RE2) participating in the classification, and the J_Bh_ distance of bands without red edge participating in the classification is the smallest. By calculating the J_Bh_ distance of different classification schemes, the study confirms that the addition of red edge band of GF-6 WFV data can improve the separability of different crop types, and the red edge 710 band is more beneficial to improving the separability of different crops than the red edge 750 band, which plays an important role in crop classification.

### 4.2. Feature Importance Evaluation Results of Red Edge Indices

**Figure 4:**
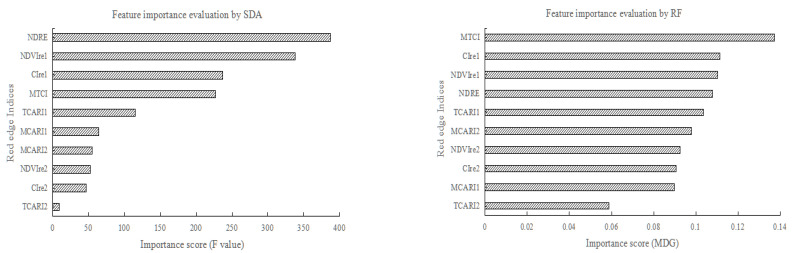
Importance evaluation results of red edge indices based on SDA and RF.

The SDA method of evaluating the importance of different features is mainly based on the F value of different features. The larger the F value, the greater the importance of the corresponding feature, and the smaller the F value, the lower the importance of the feature [67,68]. In this study, SPSS statistical analysis software [69] was used to realize the stepwise discriminant analysis and evaluation of different red edge index features. RF algorithm used random forest classifier model in the scikit-learn machine learning library of Python language to reflect feature importance by mean decrease Gini (MDG) [70,71]. The feature importance score of different red edge indices based on the two methods of SDA and RF are shown in Table 6, and the evaluation results of the feature importance of different red edge indices are shown in Figure 4.

According to Table 6 and Figure 4, although the importance order of the red edge indices obtained by the two methods was different, the top four red edge indices in the order of importance were CIre1, MTCI, NDRE and NDVIre1. Therefore, CIre1, MTCI, NDRE and NDVIre1 were selected to match the optimal red edge index 1, optimal red edge index 2, optimal red edge index 3 and optimal red edge index 4 as shown in Table 4, respectively. Combined with the four red edge indices, different classification schemes were designed to analyze the impact of different red edge indices on crop classification based on GF-6 WFV data.

### 4.3. Classification Results of Red Edge Features

**Figure 5:**
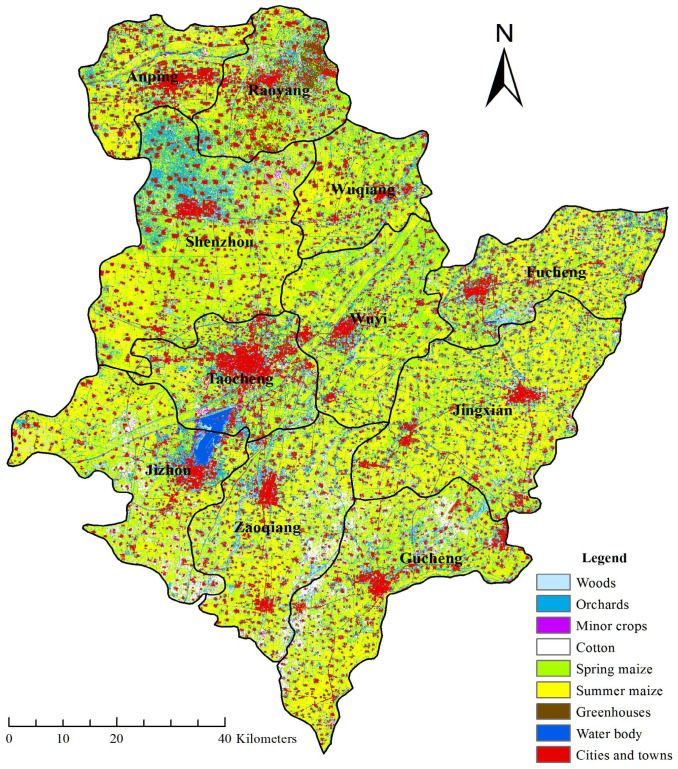
Crop classification results in Hengshui city.

**Figure 6:**
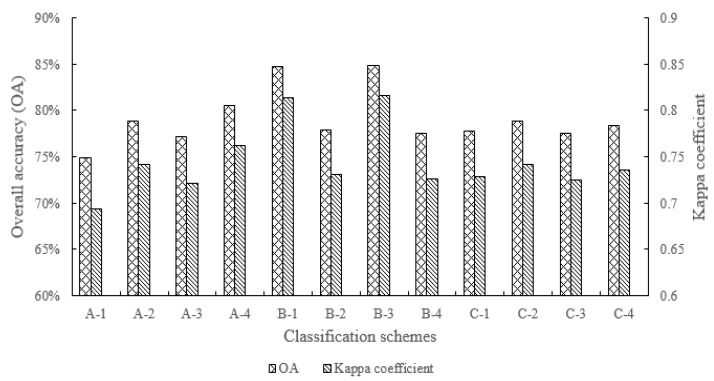
Overall classification accuracy and kappa coefficient of all different classification schemes.

Based on the RF algorithm, the classification and accuracy evaluation of 12 schemes were carried out. The crop classification results in Hengshui city are shown in Figure 5, and the OAs and kappa coefficient scores of different schemes are shown in Figure 6. The classification accuracy of different crops based on the three classification schemes of A, B and C, corresponding to different red edge spectral features, red edge texture features and red edge indices are shown in Table 7, Table 8 and Table 9. McNemar’s test results for analyses 1–23 between the two different classification schemes of red edge features are shown in Table 10.

According to the crop classification results shown in Figure 5 (taking scheme B-3 with the highest overall classification accuracy as an example), we can obtain the planting situation and spatial distribution of different crops in Hengshui City on 28 August 2019. Among them, summer maize was the most important planting type and distributed in most areas of Hengshui City. Spring maize was mainly distributed in the north and northeast of Hengshui City. Planting spring maize can save water resources and labor, which is in line with the country’s seasonal fallow policy. Cotton was mainly distributed in the southern part of Hengshui City, which was close to the cotton producing area in southern Hebei Province and was a traditional cotton planting area. Greenhouse was mainly concentrated in the east of Raoyang County in Hengshui City. Raoyang County is known as the hometown of Chinese vegetables and the hometown of Chinese facility grapes, so the distribution of greenhouses is the most concentrated in Raoyang. Orchards were mainly concentrated in the north of Shenzhou City in Hengshui City. Shenzhou City is known as the hometown of Chinese peach and fruit production base in China, so orchards are most concentrated in Shenzhou City. The minor crops in Hengshui were mainly peanuts and soybeans, as well as some peppers and potatoes, etc., which were distributed evenly and dispersed in the whole area of Hengshui City, without a large range of centralized planting and distribution.

## 5. Discussion

The results of red edge spectral analysis show that the two red edge bands and the near-infrared band of GF-6 WFV data contain more information content, among which, the red edge 750 band holds more information than the red edge 710 band, and the near-infrared band contains the most information content. The separability measure analysis of different samples shows that the red edge 710 band is more beneficial to improving the separability of different crops than the red edge 750 band, which is helpful for improving the classification accuracy of crops. The results of spectral analysis based on separability distance are consistent with the results of different red edge spectra participated in classification, and have a greater impact on crop classification than band information content.

The feature importance evaluation results of red edge indices show that CIre1, MTCI, NDRE and NDVIre1 are the top four red edge indices in SDA and RF algorithm. Combined with the crop classification results of the RF algorithm with four different red edge index features, the RF feature importance evaluation method is more consistent with the actual classification results and is more accurate than the SDA method.

The classification results of different red edge spectral features are consistent with the spectral analysis results based on separability distance, and compared with the A-1 scheme (only including R, G, B, NIR four bands), the addition of different red edge spectral information can improve the overall classification accuracy. Among them, the red edge 710 band is more beneficial to improving the overall classification accuracy than the red edge 750 band, while both the red edge 710 band and the red edge 750 band participate in the overall classification accuracy is the highest. For specific crop types, the red edge 710 band and red edge 750 band can improve the identification accuracy of crops in varying degrees. Compared with red edge 750 band, red edge 710 band is more beneficial to the identification of summer maize, spring maize and orchards, while for minor crops and greenhouse, the red edge 750 band is more effective than red edge 710 band on crop identification.

The classification results of four different texture features show that the OA and kappa coefficient of red edge 710 band texture features is better than that of red edge 750 band texture feature, and the OA and kappa coefficient of two red edge band texture features are similar to that of only red edge 710 band texture feature, while the classification accuracy of only near-infrared band texture feature is lower. For specific crop types, the texture features of red edge 710 band and red edge 750 band can improve the identification accuracy in varying degrees. Compared with the texture feature of red edge 750 band, the texture feature of red edge 710 band can improve the identification accuracy of summer maize, cotton and orchards, while the red edge 750 band texture feature is more effective for the identification of spring maize, minor crops and greenhouse.

For the classification results of different red edge index features, the order of the effect of four optimal red edge indices on the overall classification accuracy of crops in the study area is as follows: MTCI > NDVIre1 > CIre1 > NDRE, but the overall accuracies of the four different red edge indices have little difference between each other, while the red edge index based on red edge 710 band is more beneficial to improving the accuracy of crop classification than the red edge 750 band. For specific crop types, compared with the other three red edge indices, the CIre1 index is more conducive to the identification of summer maize and orchards, the MTCI index is more conducive to the identification of minor crops and woods, the NDRE index is more conducive to the identification of cotton and greenhouse, while the NDVIre1 index is more conducive to the identification of spring maize. In addition, the evaluation results of feature importance obtained by the RF algorithm are more consistent with the actual classification accuracy results, which also proves that the RF algorithm is better than the SDA method in the two different feature importance evaluation methods.

Additionally, this study compared whether there is a significant difference in the classification accuracy between different red edge feature classification schemes. Through McNemar’s test, all the different classification schemes listed (Analyses 1–23) in Table 10 have significant differences. Therefore, it can be concluded that the design of different red edge feature classification schemes is more reasonable, and there is a statistically significant difference between the classification accuracy at a certain significance level (P), which also reflects the important role of red edge in crop classification.

Schuster et al. [17] tested the application potential of red edge band of RapidEye image in improving land use classification, and considered that the addition of red edge channel can improve the accuracy of land use and land cover classification. Immitzer et al. [19] used Sentinel-2 data to classify crops and tree species in two different study areas in Central Europe, analyzed the influence of different bands of Sentinel-2 data on crop classification, and obtained that red edge bands make a certain contribution to the improvement of classification accuracy. Kim et al. [21] used RapidEye images to identify and classify paddy rice and other crops, extracted the red edge spectral features and red edge texture features of paddy rice, and analyzed the impact of different red edge features on paddy rice crop classification. Compared with the red edge texture feature, the red edge spectral feature is more conducive to improving the accuracy of classification. Ustuner et al. [22] selected RapidEye images to extract three different vegetation indices, namely Normalized Difference Vegetation Index (NDVI), the Green Normalized Difference Vegetation Index (GNDVI), and the Normalized Difference Red Edge Index (NDRE), which was used for crop classification in the Aegean Sea region of Turkey. By comparing the classification results, it was found that NDRE contributed the most to the classification accuracy, which confirmed that the red edge index constructed by the red edge band was beneficial to crop classification. The abovementioned studies have confirmed that the red edge spectra, red edge textures and red edge indices related to the red edge bands of remote sensing data are important for the classification of crops and other ground objects, but the abovementioned results are not sufficient for the analysis and evaluation of different red edge features and lack of classification and comparative research between different red edge features. The purpose of this study is to make full use of the characteristics that GF-6 WFV data contain multiple red edge bands, construct a variety of different red edge spectra, red edge textures and red edge indices, and design different red edge feature classification schemes, so as to analyze the influence of different red edge features on crop classification and promote the application of different red edge features in crop classification.

The classification results of different red edge feature schemes designed in this study confirm that the spectra, textures and red edge indices of different red edge bands can improve the classification accuracy of crops to varying degrees, and the red edge texture feature has the best effect on improving the classification accuracy. At the same time, by comparing the classification accuracy of each scheme, it can be found that the spectral features, texture features and red edge indices constructed by the red edge 710 band are more conducive to improving the classification accuracy of crops than the red edge 750 band, which is of great significance for the application of different red edge band features in crop classification. In summary, this study shows the important role of the red edge bands of GF-6 WFV data for crop identification and classification.

## 6. Conclusions

This study extracted a variety of different red edge spectra, red edge textures and red edge indices from GF-6 WFV data. By using the ABS and J_Bh_ distance method, the spectral characteristics of the red edge bands were analyzed, and it was found that the two red edge bands had a large amount of information content and were beneficial to improving the separability of different crops. Through the two feature evaluation methods of SDA and RF, this study selected four red edge indices, namely, CIre1, MTCI, NDRE and NDVIre1, with greater importance. Different red edge spectral features, red edge texture features and red edge indices can improve the classification accuracy of crops to different degrees, and there are significant differences in statistics between the classification accuracy at a certain significance level by McNemar’s test. The red edge feature extracted from red edge 710 band can improve the classification accuracy of crops more effectively, which is helpful to the application of red edge feature in crop classification. The red edge features of GF-6 WFV data are fully exploited and analyzed in this study, and the application potential of different red edge features for improving the accuracy of crop classification is discussed, which promotes the popularization and application of GF-6 WFV and other satellite data with red edge bands in the field of agricultural remote sensing monitoring.

## Figures and Tables

**Table 1:** Main information parameters of GF-6 WFV data.

Band Number	Band Name	Central Wavelength (nm)	Wavelength Range (nm)	Calibration Coefficient in 2019	Spatial Resolution (m)
B1	Blue (B)	485	450–520	0.0705	16
B2	Green (G)	555	520–590	0.0567	
B3	Red (R)	660	630–690	0.0516	
B4	Near-infrared (NIR)	830	770–890	0.0322	
B5	Red edge 1 (RE1)	710	690–730	0.0532	
B6	Red edge 2 (RE2)	750	730–770	0.0453	
B7	Purple (P)	425	400–450	0.0786	
B8	Yellow (Y)	610	590–630	0.0585	

**Table 2:** Number of samples for different classes.

Type	Training Samples		Validation Sample		Total
	Number of Polygons	Number of Pixels	Number of Polygons	Number of Pixels	Number of Polygons
Summer maize	92	5855	92	4690	184
Spring maize	50	3434	50	3055	100
Cotton	35	1788	35	1721	70
Minor crops	30	415	30	388	60
Greenhouses	15	396	15	391	30
Orchards	25	871	25	855	50
Woods	20	1234	20	881	40
Cities and towns	24	5164	24	4609	48
Water bodies	16	3143	16	3465	32

**Table 3:** Red edge vegetation index based on GF-6 WFV data (10 kinds).

Red Edge Indices	Calculation Formula (GF-6 WFV)
Normalized Difference Red Edge (NDRE) [47]	$\left(\rho_{\mathrm{RE}2}-\rho_{\mathrm{RE}1}\right)/\left(\rho_{\mathrm{RE}2}{+\rho }_{\mathrm{RE}1}\right)$
Normalized Difference Vegetation Index red edge 1 (NDVIre1) [48]	$\left(\rho_{\mathrm{NIR}}-\rho_{\mathrm{RE}1}\right)/\left(\rho_{\mathrm{NIR}}{+\rho }_{\mathrm{RE}1}\right)$
Normalized Difference Vegetation Index red edge 2 (NDVIre2) [48]	$\left(\rho_{\mathrm{NIR}}-\rho_{\mathrm{RE}2}\right)/\left(\rho_{\mathrm{NIR}}{+\rho }_{\mathrm{RE}2}\right)$
Chlorophyll Index red edge 1 (CIre1) [49]	$\rho_{\mathrm{NIR}}/\rho_{\mathrm{RE}1}-1$
Chlorophyll Index red edge 2 (CIre2) [50]	$\rho_{\mathrm{NIR}}/\rho_{\mathrm{RE}2}-1$
Modified Chlorophyll Absorption Ratio Index 1 (MCARI1) [51]	$\left[\left(\rho_{\mathrm{RE}1}-\rho_{R}\right)-0.2\left(\rho_{\mathrm{RE}1}-\rho_{G}\right)\right]\times \left(\rho_{\mathrm{RE}1}/\rho_{R}\right)$
Modified Chlorophyll Absorption Ratio Index 2 (MCARI2) [52]	$\left[\left(\rho_{\mathrm{RE}2}-\rho_{R}\right)-0.2\left(\rho_{\mathrm{RE}2}-\rho_{G}\right)\right]\times \left(\rho_{\mathrm{RE}2}/\rho_{R}\right)$
Transformed Chlorophyll Absorption Reflectance Index 1 (TCARI1) [52]	$3\left[\left(\rho_{\mathrm{RE}1}-\rho_{R}\right)-0.2\left(\rho_{\mathrm{RE}1}-\rho_{G}\right)\right]\times \left(\rho_{\mathrm{RE}1}/\rho_{R}\right)$
Transformed Chlorophyll Absorption Reflectance Index 2 (TCARI2) [51]	$3\left[\left(\rho_{\mathrm{RE}2}-\rho_{R}\right)-0.2\left(\rho_{\mathrm{RE}2}-\rho_{G}\right)\right]\times \left(\rho_{\mathrm{RE}2}/\rho_{R}\right)$
MERIS Terrestrial Chlorophyll Index (MTCI) [53]	$\left(\rho_{\mathrm{RE}2}-\rho_{\mathrm{RE}1}\right)/\left(\rho_{\mathrm{RE}1}-\rho_{R}\right)$

ρ indicates the surface reflectance of a band of GF-6 WFV data. The subscript NIR refers to near-infrared band, RE1 refers to red edge 710 band, RE2 refers to red edge 750 band, R refers to red band and G refers to green band.

**Table 4:** Different red edge feature classification schemes.

Classification Schemes Classification Features		Scheme A-1	Scheme A-2	Scheme A-3	Scheme A-4	Scheme B-1	Scheme B-2	Scheme B-3	Scheme B-4	Scheme C-1	Scheme C-2	Scheme C-3	Scheme C-4
Traditional four bands (R,G,B,NIR)		✓	✓	✓	✓	✓	✓	✓	✓	✓	✓	✓	✓
Red edge spectral features	Red edge 710		✓		✓								
	Red edge 750			✓	✓								
Red edge texture features	Red edge texture 710					✓		✓					
	Red edge texture 750						✓	✓					
	Near-infrared texture								✓				
Red edge index features	Optimal red edge index 1									✓			
	Optimal red edge index 2										✓		
	Optimal red edge index 3											✓	
	Optimal red edge index 4												✓

**Table 5:** ABS index and ranking of each band of GF-6 WFV data.

Band Name	Band Order	ABS index	Ranking
Purple (P)	1	0	7
Blue (B)	2	383.4	6
Green (G)	3	474.8	5
Red (R)	4	556.3	4
Near-infrared (NIR)	5	2418.7	1
Red edge 710 (RE1)	6	728.3	3
Red edge 750 (RE2)	7	1732.5	2
Yellow (Y)	8	0	7

**Table 6:** Red edge indices importance score based on SDA and RF.

Red Edge Indices	F Value (SDA)	MDG (RF)
CIre1	237.268	0.111
CIre2	46.414	0.091
MCARI1	64.475	0.090
MCARI2	54.886	0.098
MTCI	227.010	0.137
NDRE	387.008	0.108
NDVIre1	337.605	0.110
NDVIre2	52.812	0.092
TCARI1	115.514	0.104
TCARI2	9.566	0.059

**Table 7:** Classification accuracy statistics of red edge spectral features.

Class	Scheme A-1			Scheme A-2			Scheme A-3			Scheme A-4		
	PA%	UA%	F1%	PA%	UA%	F1%	PA%	UA%	F1%	PA%	UA%	F1%
Summer maize	87.31	74.84	80.59	91.15	77.98	84.05	88.61	77.29	82.56	91.22	78.34	84.29
Spring maize	48.35	58.38	52.89	55.61	68.92	61.55	53.39	64.16	58.28	56.20	71.66	62.99
Cotton	84.83	83.33	84.07	87.68	85.21	86.42	87.91	85.05	86.45	91.69	86.99	89.27
Minor crops	30.93	60.30	40.88	29.64	47.92	36.62	29.38	57.29	38.84	27.32	49.53	35.21
Greenhouses	75.70	86.80	80.87	75.70	88.89	81.76	75.45	90.77	82.40	75.96	91.67	83.08
Orchards	47.72	54.91	51.06	61.05	73.31	66.62	57.54	65.51	61.26	61.17	72.14	66.20
Woods	75.60	75.94	75.76	82.41	80.94	81.66	79.80	76.08	77.89	83.20	77.24	80.11
Cities and towns	98.42	74.08	84.53	98.72	75.86	85.79	98.85	74.65	85.06	98.78	79.14	87.88
Water bodies	56.94	97.77	71.96	61.15	98.24	75.37	58.07	97.91	72.90	68.40	97.97	80.56
OA (%)	74.95			78.84			77.15			80.55		
Kappa coefficient	0.6937			0.7414			0.7208			0.7627		

**Table 8:** Classification accuracy statistics of red edge texture features.

Class	Scheme B-1			Scheme B-2			Scheme B-3			Scheme B-4		
	PA%	UA%	F1%	PA%	UA%	F1%	PA%	UA%	F1%	PA%	UA%	F1%
Summer maize	90.64	77.87	83.77	88.17	77.17	82.30	90.15	78.57	83.96	88.06	75.15	81.09
Spring maize	53.85	67.01	59.71	55.35	65.42	59.96	56.01	69.05	61.85	47.89	60.06	53.29
Cotton	89.25	85.52	87.34	87.45	86.44	86.94	91.34	86.52	88.86	86.23	83.94	85.07
Minor crops	27.32	50.72	35.51	28.87	51.85	37.09	27.32	53.81	36.24	30.67	57.49	40.00
Greenhouses	75.19	80.33	77.67	77.24	83.66	80.32	78.52	80.79	79.64	72.63	81.61	76.86
Orchards	58.95	64.95	61.80	50.29	59.23	54.39	53.33	62.72	57.65	44.33	53.91	48.65
Woods	82.52	82.33	82.42	78.09	73.66	75.81	81.27	74.12	77.53	74.91	68.75	71.69
Cities and towns	96.92	97.77	97.34	97.94	77.71	86.66	97.03	98.07	97.55	98.24	81.09	88.84
Water bodies	99.83	97.66	98.73	65.11	97.03	77.93	99.83	97.46	98.63	72.38	98.24	83.35
OA (%)	84.71			77.95			84.90			77.56		
Kappa coefficient	0.8142			0.731			0.8167			0.7263		

**Table 9:** Classification accuracy statistics of red edge index features.

Class	Scheme C-1			Scheme C-2			Scheme C-3			Scheme C-4		
	PA%	UA%	F1%	PA%	UA%	F1%	PA%	UA%	F1%	PA%	UA%	F1%
Summer maize	90.94	77.64	83.76	91.07	76.95	83.42	89.49	76.73	82.62	90.75	77.64	83.68
Spring maize	55.29	69.39	61.54	52.83	69.27	59.94	53.62	67.60	59.80	55.58	69.19	61.64
Cotton	87.57	86.71	87.14	90.35	84.69	87.43	90.94	88.17	89.53	88.32	87.16	87.74
Minor crops	30.15	49.79	37.56	33.51	48.51	39.64	28.61	46.64	35.46	30.67	51.07	38.32
Greenhouses	75.70	86.55	80.76	74.17	90.06	81.35	78.26	90.00	83.72	73.40	87.50	79.83
Orchards	64.09	74.25	68.79	57.66	73.58	64.65	61.52	67.44	64.34	63.27	73.51	68.01
Woods	83.54	80.00	81.73	84.22	79.96	82.03	79.00	79.82	79.41	83.43	80.24	81.80
Cities and towns	98.68	73.21	84.06	98.31	76.61	86.11	98.74	73.81	84.47	98.76	74.44	84.89
Water bodies	54.86	97.84	70.30	62.97	97.54	76.53	56.25	97.89	71.45	57.89	98.00	72.78
OA (%)	77.82			78.82			77.48			78.35		
Kappa coefficient	0.7288			0.7413			0.7248			0.7354		

**Table 10:** Result of the McNemar’s test for different combination schemes of red edge features.

Analysis	Scheme 1	Scheme 2	f_12_	f_21_	χ^2^	*p*
1	A-1	A-2	6	786	768.18	<0.0001%
2	A-1	A-3	8	449	425.56	<0.0001%
3	A-1	A-4	15	1139	1094.78	<0.0001%
4	A-2	A-3	350	11	318.34	<0.0001%
5	A-2	A-4	10	354	325.09	<0.0001%
6	A-3	A-4	12	695	659.81	<0.0001%
7	B-1	B-2	1464	108	1169.68	<0.0001%
8	B-1	B-3	83	121	7.08	0.8%
9	B-1	B-4	1509	75	1583.99	<0.0001%
10	B-2	B-3	49	1143	1302.44	<0.0001%
11	B-2	B-4	352	274	9.72	0.2%
12	B-3	B-4	1542	70	1344.16	<0.0001%
13	C-1	C-2	154	355	79.37	<0.0001%
14	C-1	C-3	188	120	15.01	0.01%
15	C-1	C-4	27	134	71.11	<0.0001%
16	C-2	C-3	373	104	151.70	<0.0001%
17	C-2	C-4	248	154	21.98	0.0003%
18	C-3	C-4	65	240	100.41	<0.0001%
19	A-1	B-3	79	2075	1849.59	<0.0001%
20	A-1	C-2	12	789	753.72	<0.0001%
21	A-4	B-3	228	1100	572.58	<0.0001%
22	A-4	C-2	381	34	293.06	<0.0001%
23	B-3	C-2	1409	190	929.31	<0.0001%

## References

[1] Zhao C.J. (2014). Advances of research and application in remote sensing for agriculture. Trans. Chin. Soc. Agric. Mach..

[2] Wardlow B.D., Egbert S.L., Kastens J.H. (2007). Analysis of time-series MODIS 250 m vegetation index data for crop classification in the US Central Great Plains. Remote Sens. Environ..

[3] Chen Z.X., Ren J.Q., Tang H.J., Shi Y., Liu J. (2016). Progress and perspectives on agricultural remote sensing research and applications in China. Journal of Remote Sensing. J. Remote Sens..

[4] Thenkabail P.S. (2010). Global Croplands and their Importance for Water and Food Security in the Twenty-first Century: Towards an Ever Green Revolution that Combines a Second Green Revolution with a Blue Revolution. Remote Sens..

[5] Atzberger C. (2013). Advances in remote sensing of agriculture: Context description, existing operational monitoring systems and major information needs. Remote Sens..

[6] Hao P., Wang L., Niu Z., Aablikim A., Huang N., Xu S., Chen F. (2014). The potential of time series merged from Landsat-5 TM and HJ-1 CCD for crop classification: A case study for Bole and Manas Counties in Xinjiang, China. Remote Sens..

[7] Song J.W., Zhang Y.J., Li X.C., Yang W.Z. (2016). Comparison between GF-1 and Landsat-8 images in land cover classification. Prog. Geogr..

[8] Cai Y., Guan K., Peng J., Wang S., Seifert C., Wardlow B., Li Z. (2018). A high-performance and in-season classification system of field-level crop types using time-series Landsat data and a machine learning approach. Remote Sens. Environ..

[9] Li H.K., Wu J., Wang X.L. (2018). Object oriented land use classification of Dongjiang River Basin based on GF-1 image. Trans. Chin. Soc. Agric. Eng..

[10] Liu J., Wang L., Teng F., Yang L., Gao J., Yao B., Yang F. (2016). Impact of red edge waveband of RapidEye satellite on estimation accuracy of crop planting area. Trans. Chin. Soc. Agric. Eng..

[11] Delegido J., Verrelst J., Meza C.M., Rivera J.P., Alonso L., Moreno J. (2013). A red-edge spectral index for remote sensing estimation of green LAI over agroecosystems. Eur. J. Agron..

[12] She B., Huang J., Shi J., Wei C. (2013). Extracting oilseed rape growing regions based on variation characteristics of red edge position. Trans. Chin. Soc. Agric. Eng..

[13] Kanke Y., Tubana B., Dalen M., Harrell D. (2016). Evaluation of red and red-edge reflectance-based vegetation indices for rice biomass and grain yield prediction models in paddy fields. Precis. Agric..

[14] Qin Z.F., Chang Q., Shen J., Yu Y., Liu J.Q. (2016). Red Edge Characteristics and SPAD Estimation Model Using Hyperspectral Data for Rice in Ningxia Irrigation Zone. Geomat. Inf. Sci. Wuhan Univ..

[15] Qiu S., He B., Yin C., Liao Z. (2017). Assessments of Sentinel 2 vegetation red-edge spectral bands for improving land cover classification. ISPRS Int. Arch. Photogramm. Remote Sens. Spat. Inf. Sci..

[16] Forkuor G., Dimobe K., Serme I., Tondoh J.E. (2018). Landsat-8 vs. Sentinel-2: Examining the added value of sentinel-2’s red-edge bands to land-use and land-cover mapping in Burkina Faso. GIScience Remote Sens..

[17] Schuster C., Förster M., Kleinschmit B. (2012). Testing the red edge channel for improving land-use classifications based on high-resolution multi-spectral satellite data. Int. J. Remote Sens..

[18] Liu H.P., An H.J. (2014). Greening tree species spectrum characteristics analysis in Huhhot based on worldview-Ⅱ. J. Inn. Mong. Agric. Univ..

[19] Immitzer M., Vuolo F., Atzberger C. (2016). First experience with Sentinel-2 data for crop and tree species classifications in central Europe. Remote Sens..

[20] Liu J.Y., Xin C.L., Wu H.G., Zeng Q.W., Shi J.J. (2019). Potential Application of GF-6 WFV Data in Forest Types Monitoring. Spacecr. Recovery Remote Sens..

[21] Kim H.O., Yeom J.M. (2014). Effect of red-edge and texture features for object-based paddy rice crop classification using RapidEye multi-spectral satellite image data. Int. J. Remote Sens..

[22] Ustuner M., Sanli F.B., Abdikan S., Esetlili M.T., Kurucu Y. (2014). Crop Type Classification Using Vegetation Indices of RapidEye Imagery. ISPRS Int. Arch. Photogramm. Remote Sens. Spat. Inf. Sci..

[23] Hang S.Y., Yang L., Chen X., Yao Y. (2018). Study of typical arid crops classification based on machine learning. Spectrosc. Spectr. Anal..

[24] Wu J., Lu Y.N., Li C.B., Li Q.H. (2019). Fine Classification of County Crops Based on Multi-temporal Images of Sentinel-2A. Trans. Chin. Soc. Agric. Mach..

[25] Talukdar S., Singha P., Mahato S., Shahfahad, Pal S., Liou Y.-A., Rahman A. (2020). Land-Use Land-Cover Classification by Machine Learning Classifiers for Satellite Observations—A Review. Remote Sens..

[26] Zeraatpisheh M., Ayoubi S., Jafari A., Tajik S., Finke P. (2019). Digital mapping of soil properties using multiple machine learning in a semi-arid region, central Iran. Geoderma.

[27] Sonobe R., Yamaya Y., Tani H., Wang X., Kobayashi N., Mochizuki K.-I. (2017). Assessing the suitability of data from Sentinel-1A and 2A for crop classification. GIScience Remote. Sens..

[28] Maponya M.G., van Niekerk A., Mashimbye Z.E. (2020). Pre-harvest classification of crop types using a Sentinel-2 time-series and machine learning. Comput. Electron. Agric..

[29] Liu J., Wang L.M., Yang F.G., Yang L.B., Wang X.L. (2015). Remote sensing estimation of crop planting area based on HJ time-series images. Trans. Chin. Soc. Agric. Eng..

[30] Hao P., Tang H., Chen Z., Liu Z. (2018). Early-season crop mapping using improved artificial immune network (IAIN) and Sentinel data. PeerJ.

[31] Huang G.Q., Zhao Q.G. (2018). Mode of rotation/fallow management in typical areas of China and its development strategy. Acta Pedol. Sin..

[32] Xie H.L., Cheng L.J. (2017). Influence factors and ecological compensation standard of winter wheat-fallow in the ground water funnel area. J. Nat. Resour..

[33] Wang M., Guo B.B., Long X.X., Xue L., Cheng Y.F., Jin S.Y., Zhou X. (2020). On-orbit geometric calibration and accuracy verification of GF-6 WFV camera. Acta Geod. Cartogr. Sin..

[34] Zhang Q.Y., Li Z., Xia C.Z., Chen J., Peng D.L. (2019). Tree species classification based on the new bands of GF-6 remote sensing satellite. J. Geo-Inf. Sci..

[35] Liu C., Zhao C., Zhang L.Y. (2005). A new method of hyperspectral remote sensing image dimensional reduction. J. Image Graph..

[36] Zhang A.W., Du N., Kang X.Y., Guo F.C. (2017). Hyperspectral adaptive band selection method through nonlinear transform and information adjacency correlation. Infrared Laser Eng..

[37] Zhang Y., Guan Y.L. (2018). Hyperspectral band reduction by combining clustering with adaptive band selection. Remote Sens. Inf..

[38] Ma N., Hu Y.F., Zhuang D.F., Wang X.S. (2010). Determination on the optimum band combination of HJ-1A hyperspectral data in the case region of Dongguan based on optimum index factor and J–M distance. Remote Sens. Technol. Appl..

[39] Bruzzone L., Roli F., Serpico S.B. (1995). An extension of the Jeffreys-Matusita distance to multiclass cases for feature selection. IEEE Trans. Geosci. Remote Sens..

[40] Hao P., Wu M., Niu Z., Wang L., Zhan Y. (2018). Estimation of different data compositions for early-season crop type classification. PeerJ.

[41] Haralick R.M., Shanmugam K., Dinstein I.H. (1973). Textural features for image classification. IEEE Trans. Syst. Man Cybern..

[42] Palsson F., Sveinsson J.R., Ulfarsson M.O., Benediktsson J.A. (2014). Model-based fusion of multi-and hyperspectral images using PCA and wavelets. IEEE Trans. Geosci. Remote Sens..

[43] Zhao Y.S. (2013). Principles and Methods of Remote Sensing Application Analysis.

[44] Zhang L., Gong Z.N., Wang Q.W., Jin D., Wang X. (2019). Wetland mapping of Yellow River Delta wetlands based on multi-feature optimization of Sentinel-2 images. J. Remote Sens..

[45] Fang C.Y., Wang L., Xu H.Q. (2017). A comparative study of different red edge indices for remote sensing detection of urban grassland health status. J. Geo-Inf. Sci..

[46] Xie Q.Y. (2017). Research on Leaf Area Index Retrieve Methods Based on The Red Edge Bands from Multi-Platform Remote Sensing Data. Ph.D. Thesis.

[47] Gitelson A.A., Merzlyak M.N. (1994). Spectral reflectance changes associated with autumn senescence of Aesculus hippocastanum L. and Acer platanoides L. leaves. Spectral features and relation to chlorophyll estimation. J. Plant Physiol..

[48] Barnes E.M., Clarke T.R., Richards S.E., Colaizzi P.D., Haberland J., Kostrzewski M., Moran M.S. Coincident detection of crop water stress, nitrogen status and canopy density using ground based multispectral data. Proceedings of the Fifth International Conference on Precision Agriculture.

[49] Gitelson A.A., Gritz Y., Merzlyak M.N. (2003). Relationships between leaf chlorophyll content and spectral reflectance and algorithms for non-destructive chlorophyll assessment in higher plant leaves. J. Plant Physiol..

[50] Gitelson A.A., Keydan G.P., Merzlyak M.N. (2006). Three-band model for noninvasive estimation of chlorophyll, carotenoids, and anthocyanin contents in higher plant leaves. Geophys. Res. Lett..

[51] Daughtry C.S., Walthall C.L., Kim M.S., De Colstoun E.B., McMurtrey J.E. (2000). Estimating corn leaf chlorophyll concentration from leaf and canopy reflectance. Remote Sens. Environ..

[52] Haboudane D., Miller J.R., Pattey E., Zarco-Tejada P.J., Strachan I.B. (2004). Hyperspectral vegetation indices and novel algorithms for predicting green LAI of crop canopies: Modeling and validation in the context of precision agriculture. Remote Sens. Environ..

[53] Dash J., Curran P.J. (2004). MTCI: The MERIS terrestrial chlorophyll index. Int. J. Remote Sens..

[54] Blum A., Langley P. (1997). Selection of relevant features and examples in machine learning. Artif. Intell..

[55] Guyon I., Elisseeff A. (2003). An introduction to variable and feature selection. J. Mach. Learn. Res..

[56] Costanza M.C., Afifi A.A. (1979). Comparison of Stopping Rules in Forward Stepwise Discriminant Analysis. J. Am. Stat. Assoc..

[57] Zhang H., Li Q., Liu J., Du X., Dong T., McNairn H., Shang J. (2018). Object-based crop classification using multi-temporal SPOT-5 imagery and textural features with a Random Forest classifier. Geocarto Int..

[58] Wang N., Li Q.Z., Du X., Zhang Y., Zhao L.C., Wang H.Y. (2017). Identification of main crops based on the univariate feature selection in Subei. J. Remote Sens..

[59] Van der Linden S., Rabe A., Held M., Jakimow B., Leitão P.J., Okujeni A., Hostert P. (2015). The EnMAP-Box--A Toolbox and Application Programming Interface for EnMAP Data Processing. Remote Sens..

[60] Htitiou A., Boudhar A., Lebrini Y., Hadria R., Lionboui H., Elmansouri L., Benabdelouahab T. (2019). The performance of random forest classification based on phenological metrics derived from Sentinel-2 and Landsat 8 to map crop cover in an irrigated semi-arid region. Remote Sens. Earth Syst. Sci..

[61] He Y., Huang C., Li H., Liu Q.S., Liu G.H., Zhou Z.C., Zhang C.C. (2019). Land-cover Classification of Random Forest based on Sentinel-2A Image Feature Optimization. Resour. Sci..

[62] Cohen J. (1960). A Coefficient of Agreement for Nominal Scales. Educ. Psychol. Meas..

[63] Congalton R.G. (1991). A review of assessing the accuracy of classifications of remotely sensed data. Remote Sens. Environ..

[64] Foody G.M. (2004). Thematic map comparison: Evaluating the statistical significance of differences in classification accuracy. Photogramm. Eng. Remote Sens..

[65] Vasilakos C., Kavroudakis D., Georganta A. (2020). Machine learning classification ensemble of multitemporal Sentinel-2 images: The case of a mixed mediterranean ecosystem. Remote Sens..

[66] Li X., Chen G., Liu J., Chen W., Cheng X., Liao Y. (2017). Effects of RapidEye imagery’s red-edge band and vegetation indices on land cover classification in an arid region. Chin. Geogr. Sci..

[67] Li Q., Wang C., Zhang B., Lu L. (2015). Object-based crop classification with Landsat-MODIS enhanced time-series data. Remote Sens..

[68] Zhang H., Li Q., Liu J., Shang J., Du X., McNairn H., Liu M. (2017). Image Classification Using RapidEye Data: Integration of Spectral and Textual Features in a Random Forest Classifier. IEEE J. Sel. Top. Appl. Earth Obs. Remote Sens..

[69] Homer M.S. (2018). An introduction to secondary data analysis with IBM SPSS statistics. Educ. Rev..

[70] Pedregosa F., Varoquaux G., Gramfort A., Michel V., Thirion B., Grisel O., Duchesnay E. (2011). Scikit-learn: Machine learning in Python. J. Mach. Learn. Res..

[71] Raschka S. (2015). Python Machine Learning.

